# On the Mechanism by Which the Valves of the Heart Are Closed, and by Which the Sounds of the Heart Are Produced

**Published:** 1849-05

**Authors:** Joseph Hamernik

**Affiliations:** Prague


### RECORD OF MEDICAL SCIENCE.

### ANATOMY AND PHYSIOLOGY.

*On the Mechanism by which the Valves of the Heart are closed, and by which the Sounds of the Heart are produced.* By Dr. Joseph Hamernik of Prague.—In this paper the author recapitulates at some length the opinions of Dr. Baumgarten on the mechanism by which the valves are closed, which he considers of much importance, and with which he professes his concurrence.

The most important points in Dr. Baumgarten's views, are, that during the systole of the auricles, there is either no regurgitation, or of a very trifling amount, from the auricles into the great venous trunks. This, he conceives, is prevented by a circular arrangement of muscular fibres, observed by anatomists to surround the orifices of the veins; by the blood being impelled in the direction of the auriculo-ventricular orifice, in consequence of the greater portion of the muscular fibres of the auricle being inserted into the tendinous border of these openings; and by the *vis a tergo* of the blood; and in the right side, by the valve at the mouth of the vena cava. This latter, however, Dr. Hamernik considers to be inoperative in the adult, and only useful in the fœtus. He attaches great importance to the force of the current of blood flowing in the venous trunks, due to the alternate pressure exercised by the respiratory movements, reflux being prevented during expiration by the valves in the veins at the base of the neck; and in the vena cava inferior, he attributes a valvular action to the displacement of the liver during expiration, which diminishes the calibre of the vein at its passage through the diaphragm. Dr. Baumgarten considers the pulsatory movements observed in the healthy state of parts in the great veins, to depend on the sudden interruption of the current of blood during the auricular systole.

That the auriculo-ventricular valves are closed by the counter pressure of the ventricular blood, suddenly developed by the contraction of the *auricles.* That the cavities of the auricles and ventricles, during the heart's diastole, are distended by the continuous current from the veins; while at this period the valves are to be found floating in the blood in the form of a funnel. That the object of the auricular systole is to induce such an amount of tension in the contents of the ventricles, and of course in the blood surrounding the funnel-shaped arrangement of the valves, as to cause their rapid closure; and that in this way only can regurgitation be prevented. If the heart be removed from the body, and the auricles cut away (it is better, however, to operate with one only,) the artery obstructed by ligature, or by filling it with wax, and the cavity of the ventricle filled with a saline solution, the valve is found lying in the position above described. If then a stream of water be directed upon the valve from the height of a foot, so as to imitate the sudden contraction of the auricle, the valve is seen to close with great rapidity. If, however, an attempt be made to imitate the ventricular systole, by squeezing the ventricle with the hand, a large portion of its contents regurgitates before closure is effected.

That the closure is not due to the operation of the musculi papillaires, but that it is much facilitated by the small specific gravity of the valves, which enables them to float on the surface of the blood.

Dr. Hamernik then proceeds to make some remarks, which he considers in part deducible from the preceding.

1. It is possible that there may occur one or more systoles of the ventricles, unpreceded by any auricular action, forming what is called the "rythmus intercurrens" of the heart's action. In chronic asthma and pneumonia, the blood, powerfully propelled by the expiratory movements, may distend the auricles to such an extent, that they are unable to contract on their contents. In which circumstances, two or more systoles of the ventricles are required before the auricles can unload themselves.

2. The division by the older anatomists of the ventricles into portio auricularis and portio arteriosa, is physiologically and pathologically significant. In the former, there is a current of blood until the closing of the auriculo-ventricular valves, continuous with that of the veins. In the latter, a current is established by the ventricular systole, continuous with that of the arteries. Where there is no motion of fluid, there can be no murmur; consequently simple roughness of the mitral valve by exudation, or otherwise, will not give rise to a murmur with the first sound unless the valve be also insufficient.

3. The mechanism by which the valves of the arteries are closed, is similar to that of the auriculo-ventricular valves. Immediately on the contraction of the ventricles, the pressure of the blood attained in the large arterial trunks, acting equally in all directions, effects the closure of the semilunar valves. Their complete closure occurs contemporaneously with the end of the ventricular systole. When the ventricular diastole begins, the arterial retraction commences, and the wave of reflux from the large arteries, falls upon the valves already closed, and thus is produced the clear second sound. There is no regurgitation, which would necessarily be the case to a certain extent were the valves shut only by the returning wave of blood.

4. The first sound of the heart is occasioned by the vibration of the tense auriculo-ventricular valves, acted on by the blood propelled against them during the systole of the ventricles, and the vibration of the chordæ tendineæ. In like manner, the second sound is produced by the impulse of the blood on the semi-lunar valves already shut, and not by their closure.

5. A double or even a treble sound is sometimes heard over the ventricles, which has been ascribed to various causes, but is probably due to a double vibration of the tense auriculo-ventricular valves—just as a sail struck by the wind may emit several sounds. The same explanation is given when the phenomenon occurs with the second sound.

In contraction of the mitral orifice, there is occasionally heard a peculiar sound termed *cliquetis métallique*, or "audible heart impulse," and of which different explanations have been offered. According to the author's experience, all true heart sounds are heard by mediate or immediate auscultation only. This sound, however, is heard at a distance from the chest, and is hence presumed by him to depend on the motion imparted by the heart's systole to the surrounding elastic tissues.

6. Morbid conditions of the muscular structure of the heart, can have no effect in preventing closure of the valves.

7. As the small specific gravity of the valves is assumed to facilitate their closure, any thing which can render them specifically heavier, as fibrinous deposits, in the case of debilitated individuals whose blood is of low specific gravity, may be conjectured to interfere with their action. It is in such cases that the re-establishment of an improved condition of the blood removes the murmur, as in typhus fever, severe pneumonia, &c. On similar principles, the author adds, the bruits observed in chlorotic patients may perhaps be explained.—*Prager Viertel-jahrschrift.*

[We witnessed the experiments referred to in this paper, when performed by Dr. Hamernik, and have much pleasure in testifying to their accuracy. The experiment, by which it is shown that the auriculo-ventricular valve is closed before, and independently of the ventricular systole, is very easy of performance.

The valves, when cut out of the heart, are found to float readily on the surface of blood; and probably their specific lightness plays a part in the mechanism of their closure, at least in the human subject; at the same time, that it is far from being essential is indicated in prone animals, and in the case of a man standing on his head. The seat of chlorotic murmurs prevents our attributing them to the cause hinted at by the author.]—*London Monthly Journal.*

